# Psychological stress in adolescent and adult mice increases neuroinflammation and attenuates the response to LPS challenge

**DOI:** 10.1186/1742-2094-9-9

**Published:** 2012-01-16

**Authors:** Christopher J Barnum, Thaddeus WW Pace, Fang Hu, Gretchen N Neigh, Malú G Tansey

**Affiliations:** 1 Department of Physiology, School of Medicine at Emory University, 615 Michael Street, Atlanta, GA 30324, USA; 2 Department of Psychiatry and Behavioral Sciences, School of Medicine at Emory University, 615 Michael Street, Atlanta, GA 30324, USA; 3 Winship Cancer Institute, Emory University, 1365-B Clifton Rd NE, Suite 5100, Atlanta, GA 30322, USA

**Keywords:** inflammation, TNF, psychological stress, predatory stress, midbrain, corticosterone, hippocampus, LPS, depression, anxiety

## Abstract

**Background:**

There is ample evidence that psychological stress adversely affects many diseases. Recent evidence has shown that intense stressors can increase inflammation within the brain, a known mediator of many diseases. However, long-term outcomes of chronic psychological stressors that elicit a neuroinflammatory response remain unknown.

**Methods:**

To address this, we have modified previously described models of rat/mouse predatory stress (PS) to increase the intensity of the interaction. We postulated that these modifications would enhance the predator-prey experience and increase neuroinflammation and behavioral dysfunction in prey animals. In addition, another group of mice were subjected to a modified version of chronic unpredictable stress (CUS), an often-used model of chronic stress that utilizes a combination of stressors that include physical, psychological, chemical, and other. The CUS model has been shown to exacerbate a number of inflammatory-related diseases via an unknown mechanism. Using these two models we sought to determine: 1) whether chronic PS or CUS modulated the inflammatory response as a proposed mechanism by which behavioral deficits might be mediated, and 2) whether chronic exposure to a pure psychological stressor (PS) leads to deficits similar to those produced by a CUS model containing psychological and physical stressors. Finally, to determine whether acute PS has neuroinflammatory consequences, adult mice were examined at various time-points after PS for changes in inflammation.

**Results:**

Adolescent mice subjected to chronic PS had increased basal expression of inflammation within the midbrain. CUS and chronic PS mice also had an impaired inflammatory response to a subsequent lipopolysaccharide challenge and PS mice displayed increased anxiety- and depressive-like behaviors following chronic stress. Finally, adult mice subjected to acute predatory stress had increased gene expression of inflammatory factors.

**Conclusion:**

Our results demonstrate that predatory stress, an ethologically relevant stressor, can elicit changes in neuroinflammation and behavior. The predatory stress model may be useful in elucidating mechanisms by which psychological stress modulates diseases with an inflammatory component.

## Background

There is arguably nothing more ubiquitous than psychological stress and virtually all diseases are affected by it. To examine the relationship between chronic stress and disease, researchers often employ some version of the chronic unpredictable/mild stress (CUS/CMS) model. CUS has been used to examine depression [1] and exacerbate various inflammatory-related diseases including obesity [2], atherosclerosis [3], and Alzheimer's disease [4]. Although the types of stressors used in this model can vary considerably, stressors that challenge the organism psychologically (e.g., isolation/overcrowding), physically (e.g., cold/heat), and/or physiologically (e.g., insulin/lipopolysaccharide) are most common. While the vast majority of data indicates that psychological stress exacerbates the development and/or progression of many diseases, particularly during adolescence [5], the mechanism(s) remain unknown.

There is increasing evidence that stress increases inflammation, a known mediator of many diseases in humans and animals. For instance, patients with major depression subjected to the Trier Social Stress Test, a psychological stressor that requires participants to conduct a mental arithmetic problem and speak publically, show increased markers of peripheral inflammation, including plasma interleukin-6 (IL-6) and nuclear factor kappa B (NF-κB) DNA-binding relative to non-depressed controls [6]. Evidence that stress can increase inflammation within specific regions of the brain, however, has been limited to studies conducted in animals. Animal models of stress that elicit inflammatory responses such as interleukin-1 (IL-1) following footshock [7], tailshock [8], and immobilization [9] likely have a physical component that may induce elements such as pain and therefore cannot be considered psychological stressors. Furthermore, because of the nature of these stressors, chronic exposure has not been possible. Similarly, stressors typically used in the CUS/CMS models often include physical and or physiological stressors and therefore do not represent a model of psychological stress. Thus, long-term outcomes of chronic psychological stressors that elicit an acute neuroinflammatory response remain unknown.

Psychological predatory stress has been used by a number of researchers to examine a variety of stress related phenomena including fear [10], anxiety [11], post-traumatic stress disorder [12], and learning and memory [13]. Many of these predator-prey models employ the scent of a predator (e.g., cat, ferret, fox odor) to induce stress in a prey animal [14], whereas others have exposed prey to a live predator, which, in rodent studies, typically involves subjecting a rat or mouse to a live cat or snake [10,15]. In order to ensure that no harm comes to the prey, however, safeguards are put in place that limit the degree of interaction between the predator and prey. The result of this is that the predator-prey experience cannot be maximized. In addition, bringing in a cat or a snake can be very costly as it may require additional facilities and specialized handling that may not be readily available. In order to overcome these challenges, we have modified a predatory-prey model described by Blanchard and colleagues [16] to maximize the interaction between the predator (rat) and prey (mouse). This easily employed predator-prey paradigm allows sensory information to be transmitted at very close range without direct physical contact. We postulated that these modifications would enhance the predator-prey experience and have neuroinflammatory and behavioral consequences in prey animals.

To understand how PS affected mice of varying ages, both adolescent and adult mice were subjected to PS. Adolescent mice were subjected to chronic PS and examined for long-term behavioral and inflammatory changes that might lead to increased susceptibility in adulthood [5]. Specifically, chronically stressed mice were subsequently challenged, as young adults, with LPS to determine whether chronic PS modulated the inflammatory response to future inflammatory stimuli. In another set of experiments, the inflammatory response in previously non-stressed adult mice was examined following acute PS to examine how inflammation might be changing immediately following an acute stressor.

## Methods

### Animals

Male C57BL/6 mice were bred at Emory University from individuals originally purchased from The Jackson Laboratory (Bar Harbor, ME). Chronic stress studies were run in adolescent mice (P32-P60). Acute stress studies were run on adult mice (3-4 months). All mice were single-housed due to their proclivity to fight when housed in pairs. Adult male Long Evans rats (400+g upon arrival) purchased from Harlan (Indianapolis, IN, USA) served as our stimulus (predator) animal. Rats were single-housed in standard auto-water cages and had free access to standard lab chow (Rodent Diet 5001; Lab Diet, Brentwood, MO, USA) and water. Rats were housed in a separate room from mice. The colony room was maintained on a 12/12 hr light/dark cycle (lights on at 0700 hrs) at a temperature of 22-23°C. Animals were maintained in accordance with the guidelines of the Institutional Animal Care and Use Committee of Emory University.

### Rationale for experimental design

In the first series of experiments, adolescent mice (P32-P60) were subjected to 28 consecutive days of chronic PS or chronic unpredictable stress (CUS). While there is no consensus as to when adolescence begins and ends, it has been described in rodents to encompass days P20 thru P55+ [17]. The rationale for subjecting mice to chronic stress during the tail end of adolescence stems from observations that stress during this critical period increases susceptibility to many disease states later in life [18-22]. Furthermore, adult mice do not appear to have lasting effects following chronic stress (unpublished observations) limiting their utility in these models. In addition, a subset of mice were subjected to a modified version of CUS [3,4,23,24], an often-used model of chronic stress that exacerbates a number of inflammatory-related diseases via an unknown mechanism. While these CUS/CMS models have been effective in studying how stress is related to illness, they typically lack ethological validity that may undercut their usefulness. Thus, in the first series of experiments, we sought to determine two things: 1) whether stress (chronic PS or CUS) modulated the inflammatory response as a proposed mechanism by which behavioral deficits might be mediated, and 2) whether chronic exposure to a pure psychological stressor (PS) leads to deficits similar to those produced by a CUS model containing psychological and physical stressors. In order to determine whether PS had neuroinflammatory consequences regardless of age, adult mice were chosen for the acute experiments. To get a much broader picture of the inflammatory response to PS, we examined both adolescent and adult mice.

### Predatory stress (PS)

Prior to experimentation, both rats and mice were allowed to habituate to the testing room on two consecutive days for 30 min each day. PS involved placing a mouse inside a 5" diameter clear plastic hamster ball (Super *Pet*, Elk Grove Village, IL; material # 100079348) and then placing that ball into the center of the home cage of a large (400+ g Long-Evans) aggressive male rat for 30 min. To increase the aggressiveness of the male rat, at least 50% of the rat bedding remained dirty. During the PS session, mice were exposed to the sight/sound/smell of the rat through the holes in the hamster ball but never allowed to make direct physical contact. The hamster ball was not secured when placed inside the rat cage thereby allowing the rat to further agitate the mouse subject. For chronic studies, mice were subjected to daily PS for 28 consecutive days. To avoid familiarity and possible habituation, chronically stressed mice were paired with a different rat for each PS session.

### Chronic unpredictable stress (CUS)

Mice underwent chronic unpredictable stress twice each day (AM and PM). The following stressors were used: restraint (2 hr), restraint plus shaking on an orbital shaker (1.5 cycle/sec, 1 hr), continuous light (36 hrs), slanted cage (45°angle, overnight) fox odor (15 min), predatory stress (30 min), dirty rat bedding (1/2 cage covered in soiled rat bedding, overnight), open field (placed into 1 of 2 standard rat cages, 30 min), no bedding (overnight), and multiple cage changes (new cage every 30 min for 4 hours). While others have used some of these stressors in CUS paradigms [24,25] this specific paradigm has not, to our knowledge, been used previously. Descriptions of these stressors are presented in Table 1. For each week of CUS, mice were randomly assigned AM and PM stressors (see Table 2 for assignments). Mice were subjected to daily CUS for 28 consecutive days.

**Table 1:** Stressors utilized in the chronic unpredictable stress (CUS) group.

	Stressor	Description	Duration
	Predatory stress	*see PS procedure in methods*	30 min
	Restraint	Consisted of placing each mouse in a 50 mL conical with ample ventilating holes to allow for heat exchange. The mouse was confined but in no way compressed and was able to move its body.	120 min
	Restraint + Shaking	Consisted of placing each mouse into a well-ventilated 50 mL conical and resting that restraint tube onto a random orbital shaker at a speed of 1.5 revolutions per second.	60 min
***AM Stressors***	Fox odor	A single mouse was placed into an empty cage without bedding that contains a piece of filter paper (9 cm in diameter) with 0.2 mL of Fox odor (2, 5-Dihydro-2, 4, 5- trimethylthiazoline; 0.1% v/v) for 15 min.	15 min
	Novel environment	Mice were placed into 1 of 2 plastic tubs (30 in × 30 in) without bedding	30 min
	Multiple cage change	Consisted of replacing each mouse cage with a new cage every 30 min for 4 hours.	4 hrs

	Slanted cage	Each mouse cage was tilted to a 45 degree angle	overnight (12 hrs)
***PM Stressors***	Continuous light	Mice were exposed to continuous light	36 hrs
	Dirty rat bedding	1/2 of bedding from mouse cage was removed and replaced with soiled rat bedding	overnight (12 hrs)
	No bedding	Mouse bedding was removed from the home cage	Overnight (12 hrs)

Each day, mice were randomly subjected to an AM and PM stressor. Some stressors were chosen based on previous studies (e.g. [23,24]).

**Table 2:** Schedule of stressors for the chronic unpredictable stress (CUS) group.

		Monday	Tuesday	Wednesday	Thursday	Friday	Saturday	Sunday
**Week 1**	*am*	Fox odor	PS	Shaking+restraint	Novel environment	Restraint	PS	Multiple cage change
	*pm*	Dirty rat bedding	No bedding	Continuous light	Slanted cage	Slanted cage	Continuous light	No bedding
**Week 2**	*am*	PS	Shaking+restraint	Restraint	Multiple cage changes	Novel environment	Fox odor	PS
	*pm*	Slanted cage	Continuous light	Slanted cage	No bedding	Dirty rat bedding	Continuous light	No bedding
**Week 3**	*am*	PS	Shaking+restraint	Novel environment	PS	Multiple cage change	Fox odor	Shaking+restraint
	*pm*	Continuous light	Dirty rat bedding	No bedding	Slanted cage	No bedding	Continuous light	Slanted cage
**Week 4**	*am*	Fox odor	PS	Novel environment	Fox odor	Multiple cage changes	Shaking+restraint	PS
	*pm*	Continuous light	No bedding	Dirty rat bedding	Continuous light	Slanted cage	No bedding	Dirty rat bedding

### Behavioral tests

#### Marble-burying test

To measure the extent that mice subjected to either CUS or PS develop anxiety-like behavior, we utilized the marble-burying test [26]. Mice were placed in a plastic tub (50.5 × 39.4 × 19.7 cm) that contained 6" of lightly pressed bedding. Within each tub 20 marbles were evenly arranged in 5 rows of 4. The mouse was placed into the cage for 30 min after which the number of marbles covered with at least 2/3's bedding was counted.

#### Sucrose preference test

The sucrose preference test is often used as a measure of anhedonia in rodents [27]. Mice were given a choice between two bottles, one with tap water and another with 2% sucrose solution, for 48 hours. To prevent a side preference, the bottles were switched after 24 hrs. At 24 and 48 hrs, an observer blind to its contents weighed each bottle. Mice were not food or water deprived prior to experimentation.

#### Tail suspension test

The tail suspension test is commonly used to measure depressive-like behavior in mice [28]. Mice were attached to a horizontal bar suspended 30 cm above the countertop by the tail using adhesive tape and video recorded. Six minutes later, mice were returned to their home cage. All trials were videotape and later scored for latency to immobility and total time spent immobile by an observer blind to treatment condition.

### Real time quantitative polymerase chain reaction (qPCR)

Real-time quantitative RT-PCR (qPCR) was performed as previously described with some modifications [29,30]. Mouse tissue was homogenized using the TissueLyser II (Qiagen) and TRIzol^® ^Reagent (Invitrogen). To extract RNA, homogenated samples were run through Qiagen QIAshredder™ columns and then processed according to the manufacturer's instructions using Qiagen's (Valencia, CA, USA) RNeasy mini protocol for animal tissue. A DNase treatment (DNase I; Invitrogen) was included. Total RNA yield was determined by absorbance at 260 nm and purity was determined by 260/280 nm ratio using the NanoDrop 2000 spectrophotometer (Thermo Fisher Scientific, Inc.). RNA was reverse transcribed with 1 μg of normalized total RNA from each sample using the QuantiTect^® ^Reverse Transcription Kit (Qiagen). Quantitative qPCR was performed using an ABI Prism 7900 HT Fast Real-time PCR System (Applied Biosystems Inc., Foster City, CA) in 384 well format. Each sample was run in duplicate as a 10 μl reaction consisting of 25 ng cDNA, 5 ml SYBER green PCR Master mix (Power SYBER Green; Applied Biosystems), and 150 nM of each forward and reverse PCR primer. Relative gene expression of previously validated interleukin-1β (IL-1β; forward 5'-CAA CCAACAAGTGATATTCTCCATG-3' and reverse 5'-GATCCACACTCTCCAGCTGCA-3',) tumor necrosis factor (TNF; forward 5'-CTGAGGTCAATC TGCCCAAGTAC-3' and reverse 5'-CTTCACAGAGCAATGACTCCAAAG-3'), and CD-45 (forward 5'-TCATGGTCACACGATGTGAAGA-3' and reverse 5'-AGCCCGAGTGCCTTCCT-3') primers (Integrated DNA Technologies, Coralville, IA) were quantified using the 2^-ΔΔCt ^method as described previously [31] relative to the geometric means of GAPDH (forward 5'-CAAGGTCATCCATGACAACTTT-3' and reverse 5'-GGCCATCCACAGTCTTCTGG-3'), μ-actin (forward 5'-CATCGTGGGCCGCTCTA-3' and reverse 5'-CACCCACATAGGAGTCCTTCTG-3'), and HPRT1 (forward 5'-CCTAAGATGAGCGCAAGTTG-3' and reverse 5'-TACTAGGCAGATGGCCACAGG-3') [32]. All cDNA was stored at -20°C until time of assay.

### Inflammatory cytokines & receptors PCR array

Tissue was processed using Qiagen RNeasy mini kit. As described previously [33], reverse transcription was carried out using SABiosciences RT^2 ^First Strand Kit. Quantitative real-time PCR was performed using an ABI Prism 7900 HT Fast Detection System (Applied Biosystems). Each 10 μl reaction was performed in 384-well format of Mouse Inflammatory Cytokines and Receptors RT^2 ^Profiler PCR Array (SABiosciences; catalog #PAMM-011).

### Measurement of plasma corticosterone (CORT)

Plasma CORT was measured using the enzyme-immunoassay (EIA) kits from Assay Designs (Ann Arbor, MI) according to the manufacturer's instructions. The lower limit of detection was 27 pg/mL. Inter-assay coefficient was 9.21%.

### Lipopolysaccharide (LPS) treatment

Two weeks after chronic PS, mice were injected intraperitoneally (i.p.) with 7.5 × 10^5 ^EU/kg LPS [34] from Escherichia coli O111:B4 (Sigma-Aldrich, L4391) suspended in saline or Vehicle (saline).

### Statistical analysis

One-way analysis of variance (ANOVA) was used to analyze plasma CORT and mRNA in mice subjected to acute PS. Paired *t*-tests were used to examine potential habituation in plasma CORT in chronic PS experiments. For behavioral tests, a one-way ANOVA was used to assess differences in the marble burying, sucrose preference, and tail suspension test whereas a two-way ANOVA was used to examine potential differences in mice subjected to stress and LPS. Tukey's post hoc test was used where applicable. Data were analyzed with GraphPad Prism 5 (GraphPad Software, Inc., La Jolla, CA). Alpha was set at 0.05. Inflammatory Cytokines & Receptors PCR Array data was analyzed using the RT^2 ^Profiler™ PCR Array Data Analysis software on the SABiosciences website http://www.sabiosciences.com/pcrarraydataanalysis.php and are expressed as fold change. All data expressed as Mean ± standard error of the mean (SEM).

### Chronic stress

#### Experiment 1: Inflammatory response to acute LPS following chronic stress

**Figure 1:**
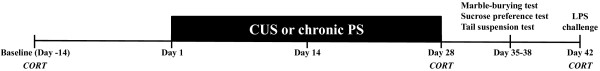
**Schematic and timeline for chronic stress**. Mice were subjected to PS, CUS, or no stress (controls) for 28 consecutive days. To determine whether chronic stress resulted in habituation of the plasma CORT response, blood was collected from the facial vein 2 weeks prior to the first day of stress (Day -14), and 15 min after stress on Day 28. For non-stressed control mice, blood was taken on same days as above. The marble burying, sucrose preference, and tail suspension test were conducted 7-10 days following the final day of stress. To determine whether chronic stress modulated the inflammatory response to a subsequent challenge, mice were injected with 7.5 × 10^5 ^EU/kg LPS at Day 42 and tissue was processed for analysis of gene expression.

To determine whether chronic stress modulates the response to a subsequent inflammatory challenge, CUS, PS, and control mice (n = 6-8/group) were injected with Saline or LPS 2 weeks after the last day of 28 days of consecutive stress (see Figure 1). Four hours after LPS or saline injection, animals were killed and midbrain and hippocampus were dissected and examined for potential changes in the expression of TNF, IL-1, and CD45 mRNA by real time PCR. Trunk blood was collected in order to examine plasma CORT by ELISA.

#### Experiment 2: Depressive and anxiety-like behaviors in mice subjected to chronic stress

Seven to ten days after the final session of PS or CUS, mice were evaluated for depressive- and anxiety-like behaviors using the sucrose preference (n = 6-9/group), the tail suspension (n = 10-16/group), and the marble burying tests (n = 9-16/group). Mice were given one test per day and tests were counterbalanced to avoid any potential influence of position and/or fatigue (see Figure 1).

#### Experiment 3: CORT response to chronic stress

Mice (n = 6/group) were subjected to chronic (28 consecutive days) PS to determine how the CORT response would change over time to daily homotypic stress. As a comparison, a subset of mice were subjected to CUS, a model of chronic stress utilized to induce depression in mice [35]. It is generally believed that the adverse outcomes of chronic stress mirror the lack of habituation of the CORT response across days, although data are sparse [36]. Thus, one of our goals was to compare how the CORT response adapts over 28 days between our PS model and the commonly employed CUS model. Plasma CORT was acquired 14 days prior to the first day of stress (baseline) and immediately after stress on the final day (day 28; Figure 1).

### Acute PS

#### Experiment 4: Acute PS as a novel, ethologically relevant psychological stressor

**Figure 2:**
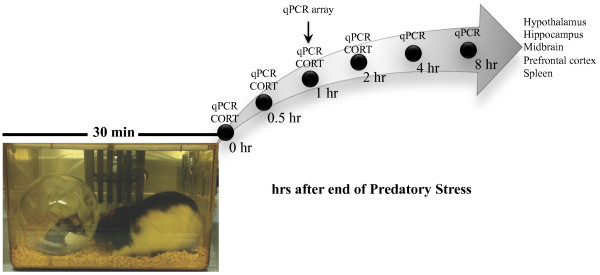
**Schematic and timeline for acute predatory stress model studies**. PS consisted of placing a mouse inside a clear plastic hamster ball and then into the home cage of a large adult male Long Evans rat for 30 min. Mice were then examined for changes in plasma CORT and inflammatory mRNA within various brain regions (hypothalamus, hippocampus, midbrain, prefrontal cortex) and spleen immediately (0 min), 0.5, 1, 2 hr after PS ended. Inflammatory mRNA was also examined at 4 and 8 hr after PS. In a separate set of mice and to determine whether PS modulated a wider number of inflammatory genes in the midbrain, a qPCR array of 84 inflammatory genes was run at 1 hr post stress.

The goals of ***Experiments 4(a-c) ***were to characterize PS as a novel, ethologically relevant psychological stressor that elicits changes in both classic indices of stress (plasma CORT) and neuroinflammation. Mice were examined for changes in plasma CORT (***Experiment 4a; ***n = 8-12/group) and inflammatory mRNA (***Experiment 4b***; n = 6-8/group) within various brain regions (hypothalamus, hippocampus, midbrain, prefrontal cortex) and spleen immediately (0 hr), 0.5, 1, 2, 4, and 8 hr after PS ended (see Figure 2). These particular structures and genes were chosen based on previous work showing their responsivity to stress and inflammation [7]. In a separate set of mice (***Experiment 4c***) and to determine whether PS modulated a wider number of inflammatory genes, midbrain was dissected and examined for potential changes in 84 inflammatory genes using SABiosciences Mouse Inflammatory Cytokines and Receptors RT^2 ^Profiler PCR Array. Samples were pooled together from 8 mice per group and run as n = 2 for both control and stressed mice.

## Results

### Experiment 1: Chronic stress increases basal inflammation in PS mice and impairs the inflammatory response to LPS challenge in both CUS and PS mice but does not differentially affect plasma CORT

Mice were subjected to 28 days of CUS or PS. Two weeks later, mice were challenged with LPS and examined for potential changes in inflammatory gene expression and plasma CORT 4 hrs later. A 2 (LPS) × 3 (stress) ANOVA was used to examine potential changes in inflammatory gene expression within the midbrain and hippocampus.

#### Midbrain (Figure 3)

**Figure 3:**
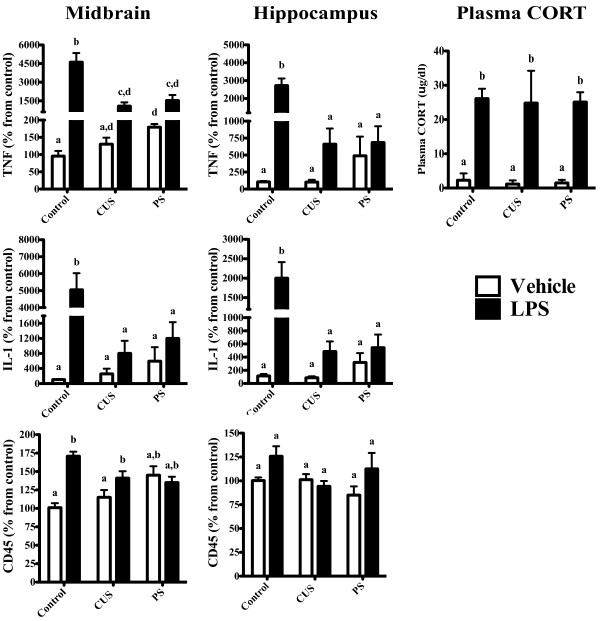
**CUS and chronic PS lead to a suppressed inflammatory response to subsequent LPS challenge**. Mice were subjected to 28 days of daily CUS or chronic PS and given an LPS challenge 14 days after the final day of stress. A 2 (LPS treatment) × 3 (Stress) ANOVA revealed that the inflammatory response to LPS was blunted (compared to controls) in mice subjected to CUS and chronic PS within the midbrain (TNF, IL-1) and hippocampus (TNF, CD45, IL-1). Within the midbrain of chronic PS mice, however, LPS did elicit a significant increase in TNF mRNA compared to chronic PS mice treated with saline (p < 0.05). A trend for an increase in IL-1 mRNA was also observed in CUS and chronic PS mice within the midbrain but did not reach statistical significance; p = 0.08 and p = 0.07, respectively. Additionally, while there was a tendency for chronic stress to increase basal levels of inflammation, this did not reach significance; midbrain (TNF & IL-1, p = 0.07) and hippocampus (IL-1, p = 0.08). Furthermore, while LPS increased plasma CORT levels in all LPS treated mice, no interaction with stress was observed. Data are expressed as percent change from control and presented as Mean ± SEM. Columns that do not share the same letter are significantly different (Two-way ANOVA p < 0.05). n = 6-8/group.

Within the midbrain, LPS increased the expression of TNF (F_1, 33 _= 44.8, p < 0.05), IL-1 (F_1, 34 _= 30.0, p < 0.05), and CD45 (F_1, 38 _= 14.7, p < 0.05) mRNA. A main effect of stress was also noted for TNF (F_2, 33 _= 10.4, p < 0.05) and IL-1 (F_2, 34 _= 11.3, p < 0.05); however, changes in CD45 mRNA as a result of stress were not observed (F_2, 38 _= 0.9, p < 0.05). A significant LPS × stress interaction was detected in all three inflammatory genes: TNF (F_2, 34 _= 11.1, p < 0.05), IL-1 (F_2, 34 _= 15.2, p < 0.05), and CD45 (F_2, 38 _= 9.5, p < 0.05). Post hoc analyses revealed a significant increase in basal levels of TNF mRNA in mice subjected to chronic PS, but not CUS, compared to controls (p > 0.05). Control mice had increased TNF, IL-1, and CD45 mRNA following LPS, an effect not observed in CUS and PS mice (p > 0.05).

#### Hippocampus (Figure 3)

Similar to what was observed in the midbrain, a main effect of LPS was detected in TNF (F_1, 33 _= 29.6, p < 0.05) and IL-1 (F_1, 32 _= 20.7, p < 0.05), but not CD45 (F_1, 35 _= 3.1, p < 0.05), mRNA. A main effect of stress was also observed in TNF (F_2, 33 _= 9.3, p < 0.05) and IL-1 (F_2, 32 _= 6.7, p < 0.05), but not CD45 (F_2, 35 _= 1.2, p < 0.05) mRNA. Finally, a significant LPS × Stress interaction for TNF (F_2, 33 _= 13.3, p < 0.05) and IL-1 (F_2, 32 _= 8.4, p < 0.05) mRNA was noted. No interaction in hippocampal CD45 was observed (F_2, 35 _= 1.6, p < 0.05). Post hoc analyses indicated that control mice injected with LPS had significantly higher TNF and IL-1 mRNA compared to saline injected controls (p > 0.05). While there was a trend for greater levels of TNF and IL-1 in LPS treated CUS mice (compared to CUS-vehicle), this did not reach statistical significance (p = 0.07). Within midbrain and hippocampus, the magnitude of the TNF and IL-1 response in control mice was significantly greater than CUS and PS mice, indicating that chronic stress may lead to a blunted inflammatory response to an immunogenic challenge.

#### Plasma CORT (Figure 3)

A 2 (LPS) × 3 (stress) ANOVA was also used to assess potential changes in plasma CORT. While LPS increased CORT levels in all mice (F_1, 23 _= 206.1, p < 0.05), no effect of stress (F_2, 23 _= 0.2, p > 0.05), nor a stress × LPS interaction was observed (F_2, 23 _= 0.0, p > 0.05), suggesting that the decrease in the inflammatory response to LPS in mice exposed to CUS/chronic PS was not driven by CORT.

### Experiment 2: Chronic psychological stress elicits long-term depressive and anxiety-like behavioral changes

#### Sucrose Preference test (Figure 4A)

**Figure 4:**
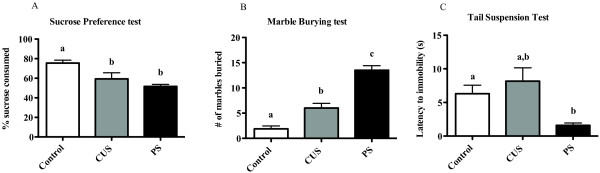
**CUS and chronic PS increase depressive-, anhedonic-, and anxiety-like behaviors**. Mice were subjected to 28 days of daily CUS or chronic PS and examined for changes in behavior 7-10 days later. A one-way ANOVA was used to examine potential differences between groups in all tests. (A) For anhedonia, mice were given a 48 hr two-bottle (water or 2% sucrose) choice to determine whether CUS or chronic PS altered sucrose preference. Control mice showed a significantly greater preference for sucrose compared to CUS and chronic PS mice. To examine whether anxiety was modulated by stress, we employed the marble-burying test. (B) Control mice showed reduced anxiety-like behavior and buried fewer marbles in a 30 min session compared to mice subjected to CUS and chronic PS mice. Additionally, chronic PS mice buried significantly more marbles than CUS mice. In a test of depressive-like behavior (tail-suspension test), chronic PS mice were faster to immobility than control and CUS mice (C). Data are expressed as Mean ± SEM. of grams of% sucrose consumed (A), number of marbles buried (B), or latency (seconds) to immobility. Columns that do not share the same letter are significantly different (One-way ANOVA; p < 0.05). Sucrose preference (n = 6-9/group), tail suspension (n = 10-16/group), marble burying (n = 9-16/group).

Mice subjected to chronic CUS and PS consumed significantly less sucrose solution than control mice (F_2, 24 _= 10.2, p > 0.05), although CUS and PS mice did not differ from each other (p < 0.05).

#### Marble-burying test (Figure 4B)

Compared to controls, increased marble-burying was observed in all mice subjected to chronic stress (F_2, 37 _= 37.8, p > 0.05). Post hoc analysis revealed that the number of marbles buried by CUS mice were significantly greater than controls but significantly less than PS mice (p < 0.05).

#### Tail suspension test (Figure 4C)

The latency to immobility in mice subjected to PS was faster than CUS and control mice (F_2, 37 _= 4.4, p > 0.05), however, there were no differences between groups in total time spent immobile (F_2, 37 _= 0.03, p < 0.05).

### Experiment 3: The CORT response attenuates in mice subjected to chronic PS and CUS by day 28

The current experiment examined whether chronic PS or CUS resulted in habituation of the CORT response in mice. Thus, CORT was assessed immediately after the final stress session (Day 28) and compared to baseline samples taken 2 weeks prior to the first day of stress. A paired *t*-test showed that plasma CORT levels on day 28 returned to baseline levels in control (*t*_5 _= 0.8, p > 0.05), CUS (*t*_5 _= 0.1, p > 0.05), and PS (*t*_5 _= 0.5, p > 0.05) mice; all plasma CORT values < 10 μg/dl (*data not presented*).

### Experiment 4a: Predatory stress is a novel, ethologically relevant psychological stressor that elicits classic stress-related responses (Figure 5)

**Figure 5:**
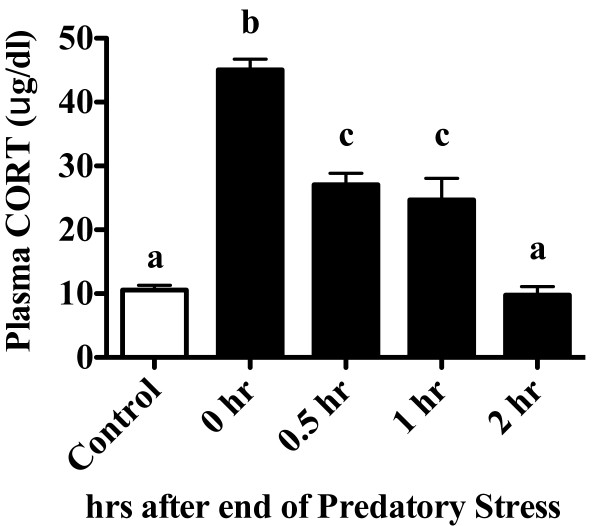
**PS elicits an increase in plasma CORT that remains elevated for an hour following cessation**. Mice were subjected to 30 min of PS and examined for changes in plasma CORT immediately (0 hr), 30 min (0.5 hr), 1 hr, or 2 hr after cessation of the stressor. A one-way ANOVA followed by Tukey's post hoc revealed that PS increased plasma CORT at 0, 0.5, and 1 hr compared to control mice and mice examined 2 hr after PS. Columns that do not share the same letter are significantly different (One-way ANOVA; p < 0.05). Data are expressed as Mean ± SEM. n = 8-12/group.

The goal of the current experiment was to characterize the CORT response to acute PS. To do this, we performed a detailed time course of the plasma CORT response in mice subjected to a single 30 min session of PS (Figure 5). Plasma was collected from mice immediately (0 hr), 30 min (0.5 hr), 1 hr, 2 hrs, 4 hrs, or 8 hrs after PS ended. Acute PS led to a time-dependent increase in plasma CORT that peaked after 30 min of PS and resolved by 2 hr (F_4, 39 _= 57.8, p < 0.0001). As depicted in Figure 5, Tukey's post hoc revealed that PS increased plasma CORT at 0, 0.5, and 1 hr compared to control mice and in mice examined 2 hr after PS.

### Experiment 4b: Acute PS leads to changes in inflammatory gene expression in multiple brain regions (Figure 6)

**Figure 6:**
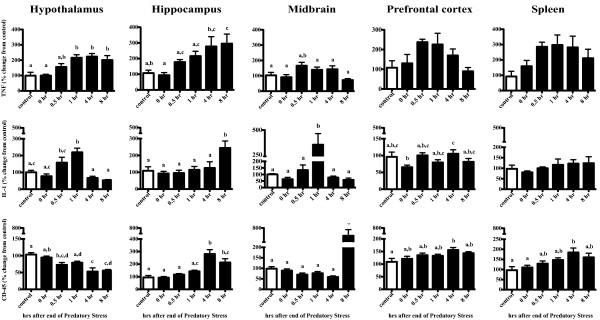
**Gene expression analyses indicate increases in Th1 cytokines and microglia activation in multiple brain regions**. To determine the extent that acute PS stimulated an inflammatory response, we conducted a time course examination of 4 brain regions and spleen. A one-way ANOVA and Tukey's post hoc revealed that both TNF and IL-1 were increased at respective time points following PS within the hypothalamus, hippocampus, and midbrain. Changes in the microglial activation marker CD45 were also increased within the hippocampus and midbrain, although not until 4 and 8 hrs after cessation, respectively. Changes in the prefrontal cortex and spleen are also provided. Columns that do not share the same letter are significantly different (One-way ANOVA; p < 0.05). Data are expressed as percent change from control and presented as Mean ± SEM. n = 6-8/group.

#### Hypothalamus

Acute PS led to an increase in both TNF (F_5, 34 _= 7.8, p < 0.05) and IL-1 (F_5, 34 _= 4.7, p < 0.05) mRNA that began 30 min after PS ended and reached peak levels at 1 hr post stress. TNF mRNA remained elevated through the 8 hr time point whereas IL-1 mRNA returned to control levels by 4 hrs. By contrast, mRNA levels of CD45, a marker of microglia activation, showed a time-dependent decrease as a result of acute PS (F_5, 34 _= 10.1, p < 0.05) such that the lowest mRNA levels were observed at the 8 hr time point.

#### Hippocampus

Mice subjected to acute PS had increased mRNA in all three genes: TNF (F_5, 33 _= 4.8, p < 0.05), IL-1 (F_5, 33 _= 4.7, p < 0.05), CD45 (F_5, 33 _= 14.5, p < 0.05). Similar to what was observed in the hypothalamus, TNF mRNA continued to increase up to the last time point examined, 8 hr; whereas increases in IL-1 mRNA were only observed at 8 hr post stress. A late increase in mRNA was also observed with CD45, which was increased at the 4 and 8 hr time point (p < 0.05).

#### Midbrain

*A*cute PS led to a time-dependent increase in TNF (F_5, 39 _= 3.5, p < 0.05), IL-1 (F_5, 39 _= 6.6, p < 0.05), and CD45 (F_5, 39 _= 4.1, p < 0.05) mRNA. TNF mRNA was increased at 0.5 hrs and IL-1 mRNA at 1 hr, whereas a change in CD45 mRNA wasn't observed until 8 hrs following PS (p < 0.05).

#### Prefrontal cortex

No significant changes in TNF mRNA were observed following PS (F_5, 31 _= 0.8, p > 0.05). While significant changes in IL-1 mRNA were noted, there was no discernable pattern that would suggest a coordinated response to acute PS (F_5, 31 _= 2.8, p < 0.05). A small but significant increase in CD45 mRNA was observed 4 hr post PS (F_5, 31 _= 3.0, p < 0.05).

#### Spleen

Neither TNF nor IL-1 mRNA were significantly altered by acute PS, (F_5, 30 _= 2.2, p > 0.05) and (F_5, 30 _= 0.7, p > 0.05), respectively. Similar to what was observed within the prefrontal cortex, CD45 mRNA was slightly elevated 4 hr after PS (F_5, 30 _= 3.9, p < 0.05), an observation that returned to controls levels by 8 hr.

### Experiment 4c: Inflammatory qPCR array analysis reveals changes in expression in multiple inflammatory genes in mouse midbrain following acute PS (Figure 7)

**Figure 7:**
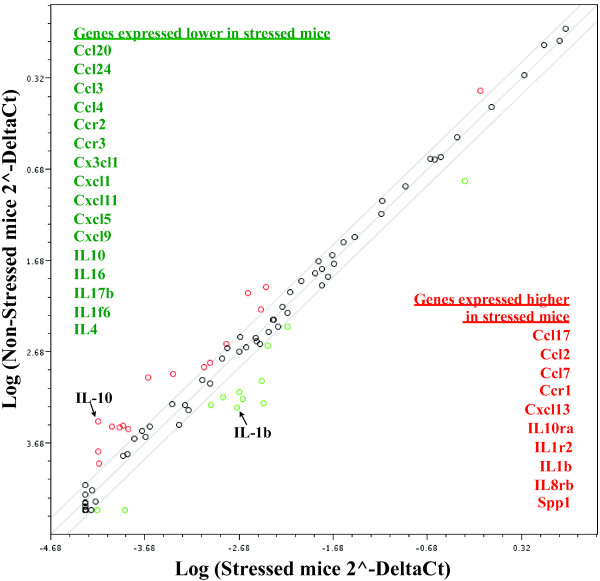
**RT-PCR analysis of inflammation-related genes reveals qualitatively different response to PS in mouse midbrain**. Mice were subjected to acute PS or remained in their home cage (control). 1 hr after acute PS, midbrain was dissected and examined for potential changes in inflammation. Samples were pooled together from 8 mice per genotype and run as n = 2 for both control and stressed mice. Mice subjected to stress show a > 2-fold increase in inflammatory factor gene expression presented in red text and > 2-fold decrease in inflammatory factors (green text) compared to control mice.

Mice were subjected to 30 min of acute PS or remained in their home cage (control). Based on results from Exp. 4b, we chose to look 1 hr after acute PS ended. Additionally, we examined the midbrain based on our previous data showing the susceptibility of the midbrain to inflammation [37]. At 1 hr post-stress, midbrain was dissected and various brain regions were used for RNA extraction; cDNA was synthesized and examined for potential changes in 84 inflammatory gene expression using the SABiosciences Mouse Inflammatory Cytokines and Receptors RT^2 ^Profiler PCR Array. Samples were pooled together from 8 mice per genotype and run as n = 2 for both control and stressed mice. In all, acute PS modulated 26 of the 84 inflammatory-related genes greater than 2-fold. Of particular interest to our group, PS led to a 6-fold increase in the mRNA for the pro-inflammatory cytokine IL-1β and 7-fold decrease in the mRNA for the anti-inflammatory cytokine IL-10. While these data suggest that a host of inflammatory-related factors may respond to acute PS, additional studies will be necessary to draw a definitive conclusion as these data reflect an n = 2 per group.

## Discussion

Although psychological stress is a harbinger for many psychiatric and medical illnesses, the biological underpinnings of this relationship remain largely unknown. The present study sought to identify potential changes in neuroinflammation in mice subjected to psychological stress. Based on previous studies examining the stress-inflammation relationship, we hypothesized that the stressor employed would have to be perceived by the animal to be significantly threatening in order to elicit a neuroinflammatory response. We therefore modified a model of predatory stress (PS) whereby a mouse is in the presence of an aggressive predator without the ability to escape. In this model, the mouse is exposed to sensory information in the absence of direct physical contact, allowing us to be confident that any response emitted by the mouse stemmed from the psychological assessment of the situation.

To verify that the PS model led to significant deficits whereby a mediator could be examined, adolescent mice were subjected to 28 consecutive days of PS. A separate cohort of mice were subjected to the often used CUS, a model of chronic stress that has been shown to elicit depressive-like behavior [1], a psychological disorder modulated by inflammation [38], and exacerbate various inflammatory-related diseases including obesity [2], atherosclerosis [3], and Alzheimer's disease [4]. Using these two stressors, we tested: 1) whether CUS/chronic PS could modulate basal expression of inflammation and subsequent response to an immunogenic challenge (LPS), 2) whether chronic PS leads to similar decrements as CUS in depressive/anxiety related behaviors and, 3) whether mice subjected to CUS/chronic PS show similar adaptation in the classic stress measures (CORT) following 28 days of stress.

Our first goal was to examine whether CUS/chronic PS could modulate basal expression of inflammation-related genes and subsequent response to a LPS challenge. Two structures were examined: the midbrain, a region we have shown to be susceptible to inflammation [39-41], and the hippocampus, a critical player in the stress response [42]. Within the midbrain of PS mice we observed ~ 2-fold increase in basal TNF mRNA compared to controls. A similar trend for an increase in basal expression of TNF (hippocampus) and IL-1 (midbrain and hippocampus) mRNA was also observed in PS mice, although this effect failed to reach statistical significance. When CUS/chronic PS mice were subsequently given LPS, the expression of TNF and IL-1 was markedly attenuated within both the midbrain and hippocampus compared to control mice, suggesting that chronic stress might facilitate organizational changes within the immune system that may lead to an inadequate response to a future stimulus.

Several researchers have looked at stress-immune interactions, although methodologies often differ considerably (see studies below). Notably, there are significant differences in regards to species and stressor used, age of the animal, duration of stress exposure (within a session and number of sessions), timing of second stimulus in relation to stress, tissue and inflammatory factors examined, as well as time after final challenge tissue is assessed. This is further complicated by the individual variability that occurs when the stress response results from interactions between two organisms in social stress situations [43,44]. Indeed, the sheer numbers of variables that exist between any two studies make comparisons difficult at best and therefore should be cautioned. That said, others have shown a decrement in responding similar to those presented herein [45]. Similarly, we have previously shown increased basal expression of IL-1 mRNA in dominant/submissive pair-housed rats. When these same rats were given acute footshock stress, we observed an attenuated IL-1 response compared to rats where a dominant/submissive hierarchy could not be determined [44]. By contrast, a number of studies have also demonstrated that stress sensitizes the inflammatory response to challenges such as LPS [46,47], an effect that has also been reported in humans [48]. The most obvious mechanism by which chronic stress might attenuate the inflammatory response is through CORT. Although CORT levels rose following acute stress, CORT levels were not different in CUS/PS mice from controls on the final day of stress and again two weeks later when mice were given LPS. Given this, and the fact that others have shown stress-induced impairment of immune function are independent of CORT [49], we suggest that other mechanism(s) that regulate cytokine expression including anti-inflammatory cytokines and intracellular signaling pathways (e.g., suppressors of cytokine signaling and NFκB) may play a more central role. Future studies will be required to assess this hypothesis.

Regardless as to whether sensitization or desensitization is observed following chronic stress, changes in a tightly regulated inflammatory system could potentially have devastating outcomes. For instance, increased basal expression of TNF and IL-1 such as those found in these studies might be enough to create a pro-death environment in susceptible brain regions (such as midbrain). In addition, the impaired cytokine response to LPS suggest that the immune response to infectious agents might be below the threshold of what is required to clear it. This in turn could have a variety of devastating effects. To this end, studies examining whether chronic PS can modulate disease in a susceptible animal are currently underway.

Increasing evidence suggests that inflammation may play a critical role in depression. This stems from initial studies showing a pro-inflammatory profile in patients that suffer from major depression (see [38] for review). As described above, researchers have used the CUS model to examine the relationship between inflammation and depression [1]. Therefore, to determine whether chronic PS, a model we have shown increases the basal expression of TNF, could also elicit behavioral deficits in disorders mediated by inflammation, depressive- and anxiety-like behaviors were assessed and compared to the CUS model. Consistent with what has been reported elsewhere, CUS led to a modest increase in anhedonia and anxiety- like behaviors [1,50-52]. PS also precipitated increased depressive-like behaviors (sucrose preference test and tail suspension test) compared to controls. The most notable effect was observed in the marble-burying test as chronic PS mice displayed considerably more anxiety-like behaviors than both control and CUS mice. Specifically, chronic PS mice buried on average 14 out of 20 marbles, whereas CUS and control mice buried an average of 6 and 2, respectively. This particular difference in anxiety-like behavior is novel as previous studies have found that CUS has little effect in eliciting an anxiogenic response in C57/BL6 mice [51,52], though it should be noted that anxiety was assessed using different tests. Neither CUS and chronic PS mice spent a greater amount of total time immobile in the tail suspension compared to controls, although this is likely due to a ceiling effect as total time spent immobile was ~330 s out of a total of 360 s for all groups. Our finding is not surprising as the C57BL/6 strain is known for their high levels of immobility in the tail suspension test [53]. Collectively, these behavioral data imply that chronic PS is at least as effective as CUS for inducing depression and may have unique utility for inducing anxiety, two disorders that are often co-morbid.

Finally, we addressed the widely accepted belief that repeated exposure to the same stressor results in a decrease in the CORT response (habituation) whereas repeated exposure to different stressors does not [36]. However, after 28 consecutive days of stress, the CORT response to both PS and CUS had returned to baseline levels. Given that CORT was not assessed after every session, the time for CORT to return to baseline levels in CUS and chronic PS mice is unclear. Despite this, Figueiredo and colleagues [54] found that the CORT response was not resolved after 14 days of predatory-prey (cat/rat) stress. Taken together, these data suggest that CORT may not be the driving factor governing the changes in inflammation and behavior following CUS and chronic PS for two reasons: 1) the total CORT response to both chronic stress models habituate by the final day of stressor exposure and 2) CUS/chronic PS and control mice had similar levels of CORT in response to Vehicle or LPS 2 weeks after the final day of stress.

It is important to note that outcomes between CUS and chronic PS mice are highly influenced by the nature of the stressors imposed upon the mice. Encounters with a predator are likely to result in wounding and or death. In order to keep the organism alive, a preparatory inflammatory response would be initiated to deal with potential wounding, infection, etc. Indeed, previous studies have reinforced this idea, demonstrating that immune cells migrate to areas close to the surface in order to quickly deal with impending injury following stress [46]. Had PS not been included in the CUS model, it is possible that inflammatory differences between PS and CUS mice might have been greater. Therefore, when examining potential inflammatory consequences of chronic stress, model selection will likely be a critical factor, and we propose the PS model is best suited for these types of analyses.

In order to more fully understand our PS model, we conducted a second series of experiments whereby the inflammatory response was examined following a single acute session of PS. For these studies, adult mice were used in order to determine whether PS has inflammatory consequences for mice of all ages. In these studies, we expanded the number of structures to include the midbrain, hippocampus, hypothalamus, prefrontal cortex, and spleen. These structures were chosen based on prior work showing their responsiveness to stressful stimuli [7,55]. Our data demonstrate that PS increased the expression of TNF and IL-1 in hypothalamus, hippocampus, and midbrain while having little effect in the prefrontal cortex and spleen. The effects of stress on IL-1 have been shown by others [7-9], although few, if any, studies have found changes in TNF following stress. Of note, CD45 mRNA was increased by stress but not until 8 hrs after PS ended. Often used as a marker for microglial activation [56], our data may indicate delayed immune cell activation. One possibility is that the increase in the expression of TNF and IL-1 observed shortly after PS facilitated the activation of microglia hours later. In this scenario, microglia would then be primed to increase the output of inflammatory factors should the organism encounter another stressful stimulus. Although this particular hypothesis is inconsistent with what we observed with chronic PS, others have shown that acute stress can potentiate an inflammatory response whereas chronic stress impairs it [57]. When and how the inflammatory response goes from sensitization following acute stress to desensitization following chronic stress is a critically important question that remains unresolved.

Taken together, our results demonstrate that PS, an ethologically relevant stressor, can elicit changes in neuroinflammation and behavior that are comparable, in some measures, and greater in others, to CUS. We further propose that the PS model may be useful in elucidating mechanisms by which psychological stress modulates diseases with an inflammatory component. The significance of psychological stress being an effector of inflammation in the brain has far-reaching implications for neurological diseases with an inflammatory component.

## List of abbreviations used

CORT: corticosterone; CUS: chronic unpredictable stress; IL-1/6: interleukin-1/6; LPS: lipopolysaccharide; PS: predatory stress; TNF: tumor necrosis factor.

## Competing interests

The authors declare that they have no competing interests.

## Authors' contributions

CJB contributed to all aspects of the paper; conceiving the project, running the assays, analyzing the data, running the stats, and writing and editing the paper. TWWP contributed to conceiving the project, analyzing the data, and editing the paper. FH contributed to running the assays and editing the paper. GNN contributed to conceiving the project, analyzing the data, and editing the paper. MGT contributed to conceiving the project, analyzing the data, and editing the paper. All authors read and approved the final manuscript.
